# Case of Lactation in a Male

**Published:** 1850-08

**Authors:** C. W. Hornor

**Affiliations:** Philadelphia


*Case of Lactation in a Male.* By C. W. Hornor, M. D., of Philadelphia. (Communicated by Professor Dunglison.)

Dear Sir,—According to your request, I send the particulars of the case of lactation in an adult male. It occurred in the person of an athletic American, named Charles Collins, aged 22 years, a blacksmith, working at his trade in New York. About the 10th of February last, his attention was first drawn to his left breast, which appeared to be enlarging, and continued to increase in size for three weeks, when he came to Philadelphia. After being in this city for three weeks, he became quite anxious in regard to his condition, for although he suffered very little pain, the mamma had become quite as large as that of a female nursing. He therefore, through the persuasion of an aunt, was, on the twenty-third of March, induced to apply at the Clinic of the Jefferson Medical College to consult the faculty of that Institution. His case came up before Prof. Mütter, who, upon examination, found the mammary gland largely developed, and filled with the lacteal secretion, which differed in no wise from that of a mother. He could assign no cause for this freak of nature; his health was very good, and the other breast natural. A soap plaster was prescribed, and compression ordered to be kept up, which he persisted in for full six weeks, when the gland returned to its usual size; and when I saw him this morning at Fairmount, where he now resides, it was in every respect like the other.

